# Strategies to Enhance Yield of Wet-Synthesized Hydroxyapatite Nanocrystals and Consequences for Drug-Release Kinetics

**DOI:** 10.3390/ma18235424

**Published:** 2025-12-02

**Authors:** Sylwester Krukowski, Natalia Byra, Aleksandra Adamczyk, Jakub Biały

**Affiliations:** Chair and Department of Pharmaceutical Chemistry and Biomaterials, Faculty of Pharmacy, Medical University of Warsaw, Banacha 1, 02-097 Warsaw, Poland

**Keywords:** hydroxyapatite, synthesis, yield, calcium source, release profile

## Abstract

Efficient production of nanocrystalline hydroxyapatite with desirable properties is a key focus in research on nanomaterials based on this mineral. It has been shown that, in the widely used wet synthesis method, fundamental factors such as the choice of calcium source and the type of post-reaction suspension maturation can play a crucial role. This study examines the influence of these two factors on parameters including synthesis yield, crystal size and morphology, material crystallinity and specific surface area, variations in OH group content, and the Ca/P molar ratio. The effect of these factors on drug release from tablets containing the synthesized hydroxyapatites was also evaluated. The results clearly demonstrate that calcium acetate decreases the synthesis yield of hydroxyapatite nanocrystals, particularly under dynamic maturation conditions. Moreover, this calcium source exerts the strongest influence on the drug release profile from tablets incorporating the obtained hydroxyapatite.

## 1. Introduction

Hydroxyapatite (HA), Ca_10_(PO_4_)_6_(OH)_2_, is an inorganic material from the calcium phosphate group that has attracted significant scientific interest for many years due to its numerous modification possibilities and applications [1]. HA materials can be ionically modified through ionic substitutions, thereby acquiring new properties [2,3]. They are also frequently used to create bi- or multiphase connections with other calcium phosphates [4,5], as well as to form composite and hybrid materials with various organic, inorganic, and metallic compounds [6,7,8,9]. The potential applications are vast and include the production of various materials, such as flexible non-flammable materials [10,11], efficient adsorbents for heavy metal ions and other pollutants [12,13], fluorescent materials [14,15], materials with catalytic properties [16,17], and materials for biomedical applications (implantology, aesthetic medicine, drug carriers) [18,19,20,21].

There are many different methods for obtaining HA materials, with wet methods, particularly classical precipitation, being the most dominant. They are the most widely used, especially for producing nanocrystalline samples. The advantages of the precipitation method include its simplicity, speed, low cost, and the fact that it does not require access to specialized equipment or the use of toxic reagents [18,22,23]. When synthesizing HA using the precipitation method, a wide range of parameters must be considered, including the selection of calcium and phosphorus sources and their concentrations, the rate of dripping and mixing, temperature, and the maturation time of the precipitate. The influence of most of these parameters on the resulting material is already well known and has been described in numerous articles [24,25,26].

The aim of this study was to determine the effect of the calcium source and the type of precipitate maturation on the yield of nanocrystalline HA and its other parameters, such as specific surface area, crystal size and morphology, crystallinity, changes in OH group content, and the Ca/P molar ratio. In precipitation methods, the three most commonly used water-soluble calcium salts are chloride, nitrate, and acetate. As for the maturation of freshly precipitated sediment, it can be dynamic (constant mixing) or static (the stirrer is turned off immediately after synthesis). In the literature, various aspects of using the mentioned calcium salts for HA synthesis can be found [27,28,29,30,31,32,33,34,35,36], but their effect on the synthesis yield has not been described so far. The same applies to the type of maturation (dynamic/static). Its influence on both the synthesis yield and the parameters of the resulting material is not known. To translate the obtained results into an application-oriented, pharmaceutical context, the synthesized hydroxyapatite powders were used as a filler material for the preparation of ibuprofen tablets. Ibuprofen was selected as a widely used model drug substance. Ultimately, the aim was to determine the influence of the hydroxyapatite synthesis method on the in vitro drug release profile.

## 2. Results and Discussion

### 2.1. Synthesis Efficiency and Specific Surface Area

**Figure 1:**
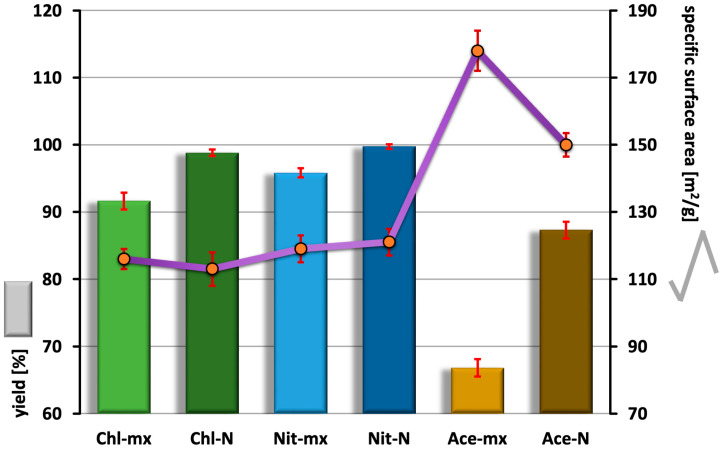
Synthesis yields (bars, left scale) and specific surface area (curve, right scale). Error bars as ±SD of three replicates.

The synthesis yields presented in Figure 1 clearly show that the use of acetate as a calcium source, combined with dynamic maturation, results in a significant decrease in synthesis yield. In contrast, simply changing the maturation type from dynamic to static, while maintaining the same calcium source, causes the yield to increase from 66.8 ± 1.3% to 87.3 ± 1.2%. The trend of higher yield due to static maturation is also observed for chloride and nitrate. However, the differences compared to the dynamic versions for these two salts are not as significant. Both the use of chloride and nitrate for synthesis, combined with static maturation, results in high yields, around 100%. Researchers describing hydroxyapatite syntheses rarely mention their yields. However, the results we obtained are consistent with the few available studies reporting the yield of the classical precipitation synthesis, where authors indicate values in the range of 80–98% [31,37,38,39]. An exception is the aforementioned use of acetate with dynamic maturation. Such a reduction in yield has not been described so far.

**Figure 2:**
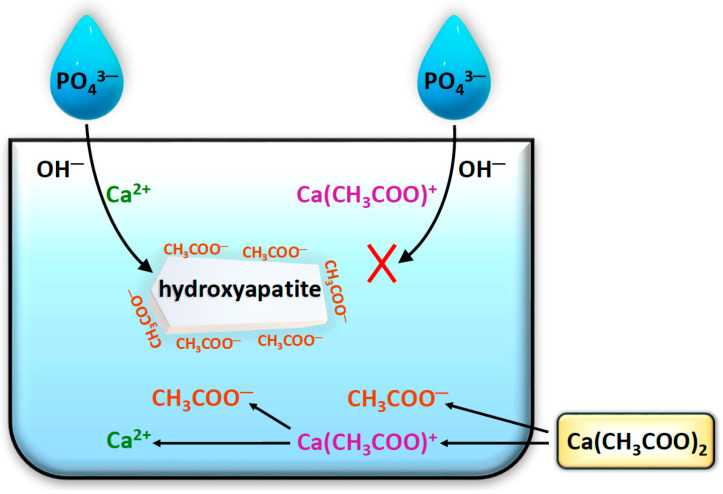
Reactions occurring during the synthesis of hydroxyapatite using calcium acetate. A red “X” indicates the absence of hydroxyapatite crystal formation.

To explain the unusual behavior of acetates, several phenomena need to be addressed. The first of these is the dissociation of calcium acetate, shown in the lower part of Figure 2. As a result, both Ca^2+^ ions (predominantly), which can precipitate HA, and ion pairs Ca(CH_3_COO)^+^ (logK = 1.18) are formed, effectively “blocking” Ca^2+^ ions [27,33]. The Ca(CH_3_COO)^+^ complexes are stable enough to partially, yet effectively, prevent the formation of HA, and this is one possible cause of the decreased yield. It is worth noting that among the calcium salts used, only acetates exhibit the ability to hydrolyze in aqueous solution: CH_3_COO^−^ + H_2_O ⇌ CH_3_COOH + OH^−^, which could influence the comparison of synthesis yields between calcium sources due to changes in pH. However, the high pH of the reaction mixture eliminates this negative effect by shifting the equilibrium of the hydrolysis reaction almost completely toward the substrates, in accordance with Le Chatelier’s principle. Another phenomenon, schematically presented in Figure 2, is the adsorption of acetate anions onto the forming HA crystals. The adsorption of simple organic compounds onto HA is well known, and a common consequence is the partial inhibition of crystal growth [40]. This type of blockage can occur at an early stage of crystallization, during the formation of crystal nuclei. Thus, under certain conditions, acetates present in the solution can either prevent nucleation or inhibit its growth to such an extent that the resulting crystallites are removed during the washing stage. This is another reason for the yield reduction associated with acetates. The loss effect during washing is not easily eliminated, as some HA particles remain suspended as a colloid above the precipitate (the solution remains slightly turbid for some time). This was noticeable in the case of Ace-mx. It is also worth noting that preliminary experiments showed that attempts to mitigate this effect—such as increasing centrifugation speed, heating, salting out, or other coagulation methods—did not lead to an increase in yield.

The phenomena described above regarding acetates, combined with the mechanical force applied during dynamic maturation of the precipitate, lead to a significant decrease in synthesis yield. This suggests that some of these effects—most likely acetate adsorption on crystallites and disruptions in their nucleation or growth—are intensified by mixing. Beyond acetates, a yield reduction effect is also observed for chlorides and nitrates. This indicates that mechanical forces during dynamic maturation negatively affect HA crystal formation, ultimately reducing the overall yield.

Table 1 shows that all obtained samples have similar average molar Ca/P ratios, ranging from 1.55 to 1.61. In each case, the ratio slightly deviates from the stoichiometric value of 10/6 (1.67), but this is typical for hydroxyapatite synthesized using the wet precipitation method [41]. Therefore, variations in synthesis yield under the described conditions do not disrupt the calcium-to-phosphorus ratio in the samples, which can be considered a favorable outcome.

The variations in specific surface area (SSA) shown in Figure 1 are correlated with synthesis efficiency and are primarily dependent on the calcium source used. The smallest changes are observed for Chl-mx and Nit-mx, as well as for Chl-N and Nit-N, with SSA values of 116.3 and 119.6 m^2^/g, and 113.1 and 120.9 m^2^/g, respectively. The use of acetate as a calcium source leads to an increase in SSA, particularly in the case of Ace-mx, where it reaches the highest value (178.5 m^2^/g). This effect is likely related to the adsorption of acetate ions on the crystal surfaces during maturation, which in turn may influence the porosity of the hydroxyapatite particles.

### 2.2. TEM Observations

**Figure 3:**
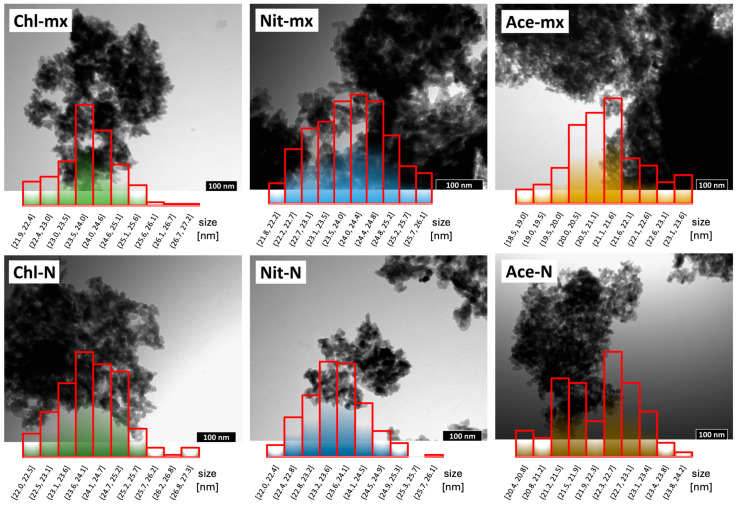
TEM images of the obtained hydroxyapatites and crystal size distribution histograms (mean values are given in Table 1).

Based on the analysis of microscopic images presented in Figure 3, it was determined that, regardless of the calcium source, the obtained samples were nanocrystalline (see Table 1 for average values) with a similar degree of aggregation. All exhibited plate-like shapes, sometimes slightly elongated, with rounded edges. The only somewhat distinctive case was Ace-mx, where the crystals were the smallest in size (21.5 nm), which is related to the previously mentioned inhibition of their growth during maturation. At the same time, the highest standard deviation (1.2 nm) is observed in this case, indicating the greatest variability in crystal size.

Due to the basic wet synthesis procedure employed—without any additives preventing aggregation—the crystals are difficult to evaluate in terms of size based on planar TEM images. Therefore, to obtain additional information regarding their dimensions, PXRD analysis was also performed.

### 2.3. PXRD Studies: Crystallinity and Crystal Size

**Figure 4:**
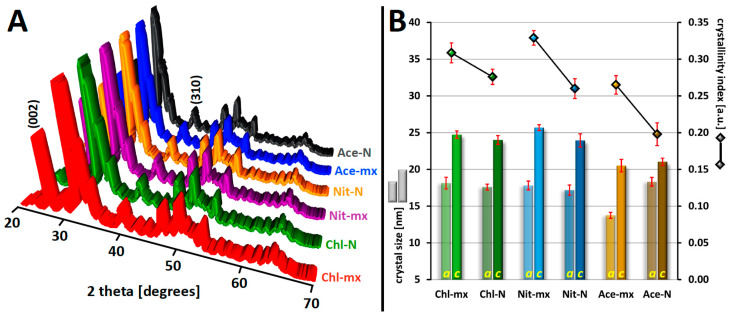
(**A**): Juxtaposition of PXRD patterns in 3D projection; (**B**): Crystal sizes along the *a* and *c* axes (bars, left scale) and crystallinity indices (square points, right scale). Error bars as ±SD of three replicates.

The diffractograms presented in a 3D projection in Figure 4A and in the conventional 2D form in Figure S1, together with the Rietveld refinement results, clearly confirm the formation of phase-pure, fine-crystalline hydroxyapatite samples. A visual analysis of the diffractograms does not reveal any significant differences between them. For this reason, computational methods were used to determine the crystallinity of the samples and the crystal sizes along the *a* and *c* axes, as described in Section 3.

The changes in sample crystallinity presented in Figure 4B are worth comparing with the synthesis yield variations shown in Figure 1. When analyzing each calcium source independently based on the type of maturation, dynamic maturation consistently results in higher hydroxyapatite crystallinity while simultaneously reducing synthesis yield. This relationship appears unusual and can be explained by the removal of the finest colloidal fraction during the washing stage. As a result, the final yield decreases, but the remaining product lacks the fraction that would otherwise contribute to lower crystallinity, as determined by computational methods. Comparing the calcium sources with each other, it can be observed that in both groups—dynamic and static maturation (Chl, Nit, Ace)—the HA synthesized using acetate has the lowest crystallinity. This trend aligns with the synthesis yield results and can be attributed to the growth-blocking effect of adsorbed acetate ions, as discussed in Section 2.1. To some extent, the HA obtained from nitrate with dynamic maturation appears to have the highest crystallinity.

The crystal sizes along the crystallographic axes *a* and *c*, presented in Figure 4B, were calculated using the Scherrer equation, considering the reflections marked in Figure 4A: (310) for the size along the *a* axis and (002) for the size along the *c* axis. The range of crystal sizes along the *c* axis (21.3–25.7 nm) is comparable to the results obtained from TEM images (21.5–24.1 nm), where the longest dimension of each crystal was considered. The relationships between the samples do not directly coincide for the two methods. However, given that the diffractogram is recorded from a powder portion substantially larger than that which can be deposited on a TEM grid, PXRD appears to provide more representative results. The results show that, regardless of the sample type, nanocrystals were obtained, with the size along the *c* axis being larger than along the *a* axis. The smallest difference in these sizes concerns Ace-N. In general, HA obtained from chloride and nitrate has crystals that are very similar in size, regardless of the maturation type. Syntheses from acetates stand out slightly in this case. Based on our results, it can be observed that these anions partially inhibit crystal growth along the *c* axis, and, in the case of dynamic maturation, also along the *a* axis. This effect may be attributed to acetate adsorption on the HA surface, which contributes to slightly smaller crystal sizes and, at the same time, results in a higher SSA.

### 2.4. FT-IR Analysis

**Figure 5:**
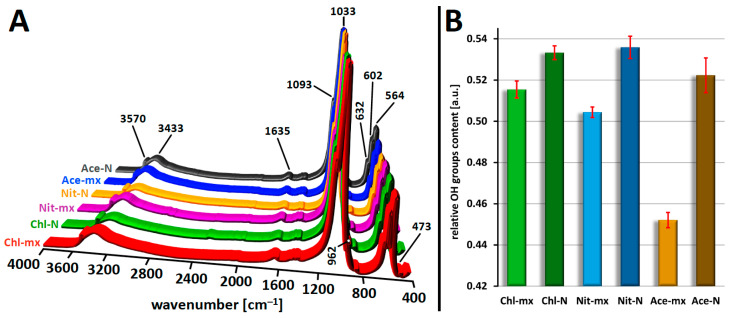
(**A**): Juxtaposition of FT-IR spectra in 3D projection; (**B**): Changes in relative contents of hydroxyapatite OH groups. Error bars as ± SD of three replicates.

The FT-IR spectra presented in a 3D projection are shown in Figure 5A, and in the conventional 2D form in Figure S2. No significant differences were observed between them. The most important bands characteristic of HA are visible, corresponding to the vibrations of phosphates: 1093 and 1033 cm^−1^ (ν_3_P–O), 962 cm^−1^ (ν_1_P–O), 602 and 564 cm^−1^ (ν_4_P–O), 473 cm^−1^ (ν_2_P–O), as well as stretching vibrations of the structural OH groups: 3570 cm^−1^ (low intensity, masked by an extensive band originating from water molecules) and 632 cm^−1^ (OH libration oscillations). There are also visible bands of covalent O–H bonds of water molecules, corresponding to bending vibrations at approximately 1635 cm^−1^ and stretching vibrations at 3200–3600 cm^−1^. No bands corresponding to nitrates and acetates were found in the spectra. In the range of 1400–1500 cm^−1^ and around 870 cm^−1^ (Figure 5A and Figure S2), additional bands of very low intensity can be observed, which are commonly detected in hydroxyapatite obtained via the classical precipitation method. As mentioned in Section 2.1, the wet synthesis method typically yields so-called non-stoichiometric hydroxyapatite, characterized by a Ca/P molar ratio lower than 1.67 (see Table 1). This may result from the presence of additional phosphate groups in the form of HPO_4_^2−^ (band at 870 cm^−1^) as well as from the frequent incorporation of CO_3_^2−^ ions (bands at 1400–1500 cm^−1^, and also at 870 cm^−1^) when synthesis is conducted under non-controlled conditions (i.e., without CO_2_ exclusion). However, since all samples were synthesized under identical conditions and the mentioned bands exhibit similar, very low intensities, a significant influence of these substitutions on synthesis efficiency can be ruled out. Additionally, considering the phase purity determined by PXRD, it can be concluded that the samples do not contain residues of the salts used in the syntheses. Therefore, they do not affect the yields.

FT-IR spectra also allow for a more detailed analysis of the composition of HA samples. One example is the comparison of the content of structural hydroxyapatite OH groups, which reflects the degree of disorder in the crystal lattice. This can be performed by extracting component bands in the 480–720 cm^−1^ range and referring to the areas of the isolated bands [42] or by using other mathematical methods, as proposed in this study. Figure 5B presents changes in the relative content of OH groups, calculated as the ratio of the height of the 632 cm^−1^ band (OH libration oscillations) to the height of the 602 cm^−1^ band (ν_4_P–O). These relationships are directly correlated with the changes in synthesis yields shown in Figure 1. The higher the yield, the greater the relative content of OH groups. In the ordered crystal lattice of hydroxyapatite, OH groups are arranged in columns, forming a kind of channel. When lattice disorder occurs, the incorporation of OH groups into regular channel positions becomes energetically less favorable, leading to the formation of HA with a lower OH content [43,44]. Therefore, the correlation between synthesis yield and the relative content of OH groups is an expected phenomenon: higher yield results in a higher OH group content. The HA samples with the fewest crystal lattice disruptions, indicated by a lower relative decrease in OH group content, are those subjected to static maturation, making this method the more favorable option. The advantage of static maturation is particularly pronounced in synthesis using acetate. When this calcium source is combined with dynamic maturation, it results in crystals that are significantly more depleted in OH groups compared to all other cases, which also corresponds to the lowest synthesis yield and the highest specific surface area. Ultimately, it can be concluded that increasing the synthesis yield leads to a reduction in specific surface area and a higher degree of crystal lattice order, as reflected in changes in OH group content. However, variations in OH group content do not correlate with disturbances in the Ca/P ratio (see Figure 1). This suggests that in HA samples depleted in OH groups, vacancies likely form in the crystal lattice. This, in turn, could have a beneficial effect, as such crystals may be more susceptible to ionic modifications (doping with ions), potentially granting them new properties.

### 2.5. Drug Release Profiles

**Figure 6:**
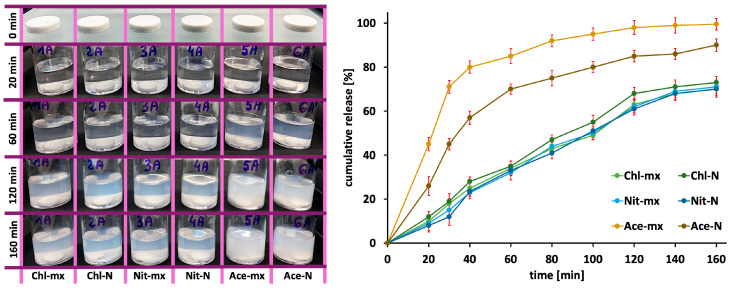
Photographs of the tablets before and during drug release (**left**) and ibuprofen release profiles from the tablets (**right**). Error bars as ± SD of three replicates.

In the drug release study, tablets containing each type of prepared hydroxyapatite were considered, and each tablet type was tested in three replicates. One of the three tablet series prior to immersion in the medium, as well as the changes in the appearance of the medium over time during the release study (for four selected time points), are shown in Figure 6. Similar trends were observed in the remaining two series (replicates).

As the drug release progressed, the medium became increasingly turbid due to tablet disintegration and the formation of suspensions. However, from approximately 60 min of release, tablets containing Ace-mx and Ace-N caused more pronounced turbidity compared to the other formulations. This effect was slightly stronger for Ace-mx from around 120 min of release. The observed degree of turbidity resulting from tablet disintegration is reflected in the drug release profiles. As shown in Figure 6, tablets containing Ace-mx and Ace-N released ibuprofen faster and in greater amounts than the others, with Ace-mx exhibiting the highest release (approximately 100% of ibuprofen released within 160 min).

For a more detailed analysis of the ibuprofen release profiles, the obtained data were fitted to the zero-order and first-order kinetic models, as well as the Higuchi model. The results of these fittings are summarized in Table 2.

Comparison of the R^2^ values for the kinetic model fittings indicates a certain predominance of first-order kinetics, particularly for tablets containing Ace-mx and Ace-N. First-order kinetics are characteristic of the release of hydrophilic drugs from hydrophilic carriers. As the release progresses, the path through which the release medium penetrates the tablet pores and through which the drug is leached becomes longer, while the concentration gradient decreases. These effects result in a decreasing release rate over time, leading to first-order kinetics. In the other cases (Chl and Nit), the differences in R^2^ values between the zero-order and first-order models are small, indicating that the release kinetics of ibuprofen cannot be unambiguously defined. At the same time, a contribution from zero-order kinetics, which is usually most desirable due to its ability to provide a uniform drug release over time, cannot be excluded.

The Higuchi model generally provides a reasonably good description of ibuprofen release from the prepared tablets; however, the fit is poorest for Ace-mx (lowest R^2^ value). Determination of the model constant (K in Table 2) allows a quantitative assessment of the drug release rate. This rate is highest for Ace-N and Ace-mx and comparatively lower in the other cases, as also reflected in the release profiles shown in Figure 6.

The release of ibuprofen is governed by two primary, concurrent processes: tablet disintegration and drug dissolution. When two excipients are used (starch and hydroxyapatite), the only variable in this case is the type of hydroxyapatite employed. Significant changes in its physicochemical properties—such as crystal size, specific surface area, and hydroxyl group content—can influence both tablet disintegration and drug dissolution, directly affecting the observed release profile. Among these parameters, SSA appears to have the most pronounced impact on drug release. Hydroxyapatites synthesized using calcium acetate, particularly Ace-mx, exhibit higher SSA and slightly smaller crystals. This likely facilitates faster migration of the dissolution medium through the tablets, thereby accelerating drug release. Simultaneously, this is reflected in a more rapid disintegration of the tablets themselves.

The results of the ibuprofen release studies, using it as a model drug, clearly demonstrate that the calcium source used during hydroxyapatite synthesis can influence not only the physicochemical properties of hydroxyapatite but also the disintegration rate of tablets containing it as a filler, and consequently the drug release rate. These findings may therefore be useful in the design of drug carriers containing hydroxyapatite. By selecting the calcium source and the method of precipitate maturation, the drug release rate can be controlled: for slower release, nitrate or chloride should be used as the calcium source (with any maturation method), whereas for faster release, acetate should be used as the calcium source and the precipitate subjected to static maturation.

## 3. Materials and Methods

### 3.1. Synthesis Procedure

The syntheses were conducted in a quantitative manner, with the only variables being the different calcium sources and the type of precipitate maturation. Therefore, solutions of the appropriate calcium salts were prepared, namely CaCl_2_ (calcium chloride hexahydrate ≥ 99%, Sigma-Aldrich, St. Louis, MO, USA), Ca(NO_3_)_2_ (calcium nitrate tetrahydrate ≥ 99%, Sigma-Aldrich), Ca(CH_3_COO)_2_ (calcium acetate monohydrate ≥ 99%, Sigma-Aldrich), and phosphates (ammonium phosphate dibasic ≥ 98%, Sigma-Aldrich), with known and precisely defined concentrations of exactly 0.8 mol/L in each case. The least soluble compound in this set is calcium acetate, whose saturated solution at 25 °C has a concentration of approximately 25%. It was assumed that the concentrations of the calcium and phosphorus sources used would be the same and roughly half of the saturation concentration of the least soluble compound. For this reason, solutions of each salt were prepared at a concentration of 0.8 mol/L. A 37.5 mL portion of each calcium salt solution was pipetted into a reaction flask, and then 30 mL of a 15% NH_3_·H_2_O solution was added. The amount of ammonia to be added was previously determined in preliminary experiments to ensure that the pH of each post-reaction mixture was the same and equal to 13, thus eliminating the need for pH control during the synthesis, which could lead to quantitative losses.

In the next step, the clear mixture of calcium salt and ammonia was stirred on a standard magnetic stirrer at 300 rpm while simultaneously adding 22.5 mL of the phosphate solution at a rate of one drop per second (theoretical Ca/P molar ratio: 1.67). At this stage, a white hydroxyapatite precipitate formed. After the entire volume of the phosphate solution was added, mixing was stopped for the static maturation samples. In contrast, for the dynamic maturation samples, stirring continued at the same speed until the maturation period ended. Regardless of the maturation type, the maturation time was set to 24 h. Throughout both the synthesis and maturation processes, the temperature was maintained at 25 °C. After maturation, the precipitates were repeatedly washed with fresh portions of water until the filtrate reached a neutral pH and a stable, low conductivity value (<1 μS, measured by a DiST5 conductivity meter, Hanna Instruments), indicating the complete removal of residual reagents. Washing was carried out by centrifuging the precipitate in containers. After washing, the containers with the hydroxyapatite precipitate were placed in a drying oven at 80 °C for 48 h. A relatively short maturation time and the shortest possible drying at a moderate temperature were chosen to focus on a fast, cost-effective, and efficient method for producing nanocrystalline hydroxyapatite. Drying the precipitates directly in the containers used for washing eliminated losses associated with transferring the reaction products to other vessels. After drying and cooling, the precipitates were carefully weighed. The preparation of each sample was repeated three times, strictly following the procedure described above, so the results of the subsequent experiments represent the averages of the three repetitions. Table 3 presents the obtained samples, taking into account the variable parameters.

The yield was calculated with respect to the theoretical amount of stoichiometric hydroxyapatite, Ca_10_(PO_4_)_6_(OH)_2_, which could be obtained based on the stoichiometry of the reaction between the employed Ca^2+^ and HPO_4_^2−^ ions in alkaline solution.

### 3.2. Physicochemical Characteristics of Hydroxyapatites

Infrared spectroscopy (FT-IR) studies were carried out at 25 °C on a Perkin Elmer Spectrum 1000 spectrometer (Waltham, MA, USA). Spectra were acquired in the 400–4000 cm^−1^ range at a spectral resolution of 2 cm^−1^ from KBr pellets using a MCT detector and 30 scans.

Powder X-ray diffraction (PXRD) experiments were carried out using a Bruker D8 Advance diffractometer (Billerica, MA, USA), having Cu Kα radiation source (λ = 1.54 Å), LYNXEYE detector and TOPAS Academic software (v.7). The degree of crystallinity was calculated using Equation (1) [45]:

(1) $$\mathrm{CI}\;={\left(\frac{K}{\beta_{002}}\right)}^{3}$$

where CI—crystallinity index, K—constant (0.24 for hydroxyapatite), and β_002_—reflection (002) full width at half maximum [degrees]. Crystal sizes were calculated using Scherrer Equation (2) [46]:

(2) $$L\;=\frac{k\cdot \lambda }{\beta \cdot \cos\theta }$$

where L—crystal size [nm], k—constant (0.94 for hydroxyapatite), λ—length of the radiation wave [nm], β—full width at half maximum [rad], and θ—the diffraction angle of the corresponding reflex [degrees].

Crystals were observed using a high-performance transmission electron microscope (TEM) JEM 1400 (JEOL Co., Kishima, Japan). A drop of the sample suspension in anhydrous ethanol was placed on a Cu/formvar grid, dried, and measured under an accelerating voltage of 80 kV. Crystal size measurements were conducted on 200 crystals from each sample using the MeasureIT software (v. 5.1, Olympus Soft Imaging Solutions GmbH). The measurements were based on the longest dimension of each crystal.

The specific surface area (SSA) of the hydroxyapatite powders was measured via physical nitrogen adsorption isotherm using the Brunauer–Emmett–Teller (BET) method (Micromeritics ASAP 2020 apparatus, Norcross, GA, USA).

The calcium and phosphorus content were determined using ICP-OES (Thermo Scientific iCap 7400 DUO spectrometer, Waltham, MA, USA) and flame photometry (BWB XP flame photometer, Newbury, UK) methods. Samples for measurement were prepared by dissolving them in ultrapure nitric acid.

### 3.3. Tablet Preparation and In Vitro Drug Release Study

The hydroxyapatite samples listed in Table 1 were prepared in larger quantities based on the procedure described above and subsequently micronized to a fine powder consistency. Tablets were produced as mixtures of hydroxyapatite, soluble starch (Sigma-Aldrich), and ibuprofen sodium salt (≥98%, Supelco, Sigma-Aldrich) in a mass ratio of 3:1:2 and were compressed using a hydraulic press under a pressure of 8 tons. Each tablet contained precisely 200 mg of ibuprofen sodium salt.

The tablets were placed in sealed containers, after which 15 mL of preheated phosphate-buffered saline (PBS, pH = 7.40 ± 0.05, Sigma-Aldrich) was added, and the containers were then shaken in an incubator at 80 rpm and 37 °C. Samples of the release medium were collected in 1 mL volumes at appropriate time intervals from 20 to 160 min. The withdrawn volume of medium was replaced with an equal amount of fresh, preheated PBS.

The amount of drug released in the collected medium samples was determined spectrophotometrically (Shimadzu UV-1800 spectrophotometer, Kyoto, Japan) using semi-micro quartz cuvettes at an analytical wavelength of 264 nm. If necessary, the collected medium samples were diluted to fall within the central range of the calibration curve, corresponding to concentrations of 68–680 mg/L.

The data obtained from the drug release study were fitted to appropriate theoretical mathematical models, including the zero-order (Equation (3)) and first-order (Equation (4)) kinetic models, as well as the Higuchi model (Equation (5)), which can be described by the following equations:

Q = Q_0_ + Kt (3)

lnQ = lnQ_0_ + Kt (4)

Q = Kt^0.5^ (5)

where Q—amount of drug released, Q_0_—initial drug concentration, t—time, and K—constant specific to the given model [47,48].

## 4. Conclusions

When planning the synthesis of hydroxyapatite using the classical wet precipitation method, in addition to basic parameters, it is also essential to consider the type of water-soluble calcium salt and determine the maturation method for the precipitate after synthesis. Our results show that these two parameters, often selected arbitrarily, have a significant impact on the synthesis yield and the physicochemical properties of the obtained hydroxyapatite.

Therefore, to achieve high synthesis efficiency, it is advisable to use calcium chloride or calcium nitrate (with a slight advantage for the latter) and to subject the freshly precipitated material to static maturation (without stirring). At the same time, due to the specific behavior of acetates, which leads to a significant decrease in synthesis yield under dynamic maturation and the formation of crystals with reduced OH group content, this calcium source should be selected with caution. This calcium source is certainly worth considering when, for specific reasons, obtaining a material with small crystal sizes and a more developed specific surface area is important.

The method of hydroxyapatite synthesis affects not only its physicochemical properties and the overall synthesis efficiency but also has practical implications for drug release from a hydroxyapatite-containing carrier. By selecting an appropriate synthesis method, drug release can be slowed when chloride or nitrate is used as the calcium source, or accelerated when acetate is used, particularly in combination with static maturation.

## Figures and Tables

**Table 1:** Ca/P molar ratios and crystal sizes determined from TEM images.

Sample Code	Theoretical Ca/P Molar Ratio	Experimental Ca/P Molar Ratio ^a^	Crystal Size [nm] ^a,b^
Chl-mx	1.67	1.55 ± 0.04	23.9 ± 0.9
Chl-N	1.67	1.55 ± 0.05	24.1 ± 1.1
Nit-mx	1.67	1.61 ± 0.05	23.9 ± 1.0
Nit-N	1.67	1.58 ± 0.06	23.6 ± 0.7
Ace-mx	1.67	1.56 ± 0.04	21.5 ± 1.2
Ace-N	1.67	1.60 ± 0.05	22.2 ± 0.8

^a^ Mean ± SD. ^b^ Based on TEM (longest dimension of each crystal), *n* = 200.

**Table 2:** Results of the mathematical model fittings to the ibuprofen release profiles from the tablets.

Sample Code	Zero-Order Kinetics	First-Order Kinetics	Higuchi Model	
	R^2^	R^2^	R^2^	K [mg/min^0.5^]
Chl-mx	0.9794	0.9872	0.9473	6.2089
Chl-N	0.9718	0.9862	0.9514	6.5459
Nit-mx	0.9844	0.9891	0.9360	6.3761
Nit-N	0.9813	0.9896	0.9298	6.3505
Ace-mx	0.6472	0.9912	0.8793	7.4598
Ace-N	0.8078	0.9709	0.9538	7.6634

**Table 3:** Descriptions of hydroxyapatite samples in relation to the synthesis parameters.

Sample Code	Calcium Source	Maturation Type
Chl-mx	chloride	dynamic (with mixing)
Chl-N	chloride	static (without mixing)
Nit-mx	nitrate	dynamic
Nit-N	nitrate	static
Ace-mx	acetate	dynamic
Ace-N	acetate	static

## Data Availability

The raw data underlying the presented data in this study will be made available upon request.

## Supplementary Materials

The following supporting information can be downloaded at https://www.mdpi.com/article/10.3390/ma18235424/s1. Figure S1: PXRD patterns in conventional form; Figure S2: FT-IR spectra in conventional form. Bands corresponding to ions typical of non-stoichiometric hydroxyapatites are highlighted in pink.

## Abbreviations

The following abbreviations are used in this manuscript:

HA	hydroxyapatite
Chl	chloride (as calcium source)
Nit	nitrate (as calcium source)
Ace	acetate (as calcium source)
-mx	dynamic maturation type
-N	static maturation type
SSA	specific surface area
BET	Brunauer–Emmett–Teller isotherm
TEM	transmission electron microscopy
PXRD	powder X-ray diffraction
FT-IR	Fourier-transform infrared spectroscopy
PBS	phosphate buffered saline
