# Supplementary material for: Strategies to Enhance Yield of Wet-Synthesized Hydroxyapatite Nanocrystals and Consequences for Drug-Release Kinetics
Source: Materials (Basel). 2025 Dec 2;18(23):5424. doi: 10.3390/ma18235424 (PMC12693289; doi:10.3390/ma18235424)
Supplement: Supplementary file 1 [file materials-18-05424-s001.zip › materials-3957654-supplementary.pdf]

materials

Supplementary Materials

Strategies to Enhance Yield of Wet-Synthesized Hydroxyapatite Nanocrystals and Consequences for Drug-Release Kinetics

Sylwester Krukowski *, Natalia Byra, Aleksandra Adamczyk and Jakub Biały

Chair and Department of Pharmaceutical Chemistry and Biomaterials, Faculty of Pharmacy, Medical University of Warsaw, Banacha 1, 02-097 Warsaw, Poland
* Correspondence: sylwester.krukowski@wum.edu.pl

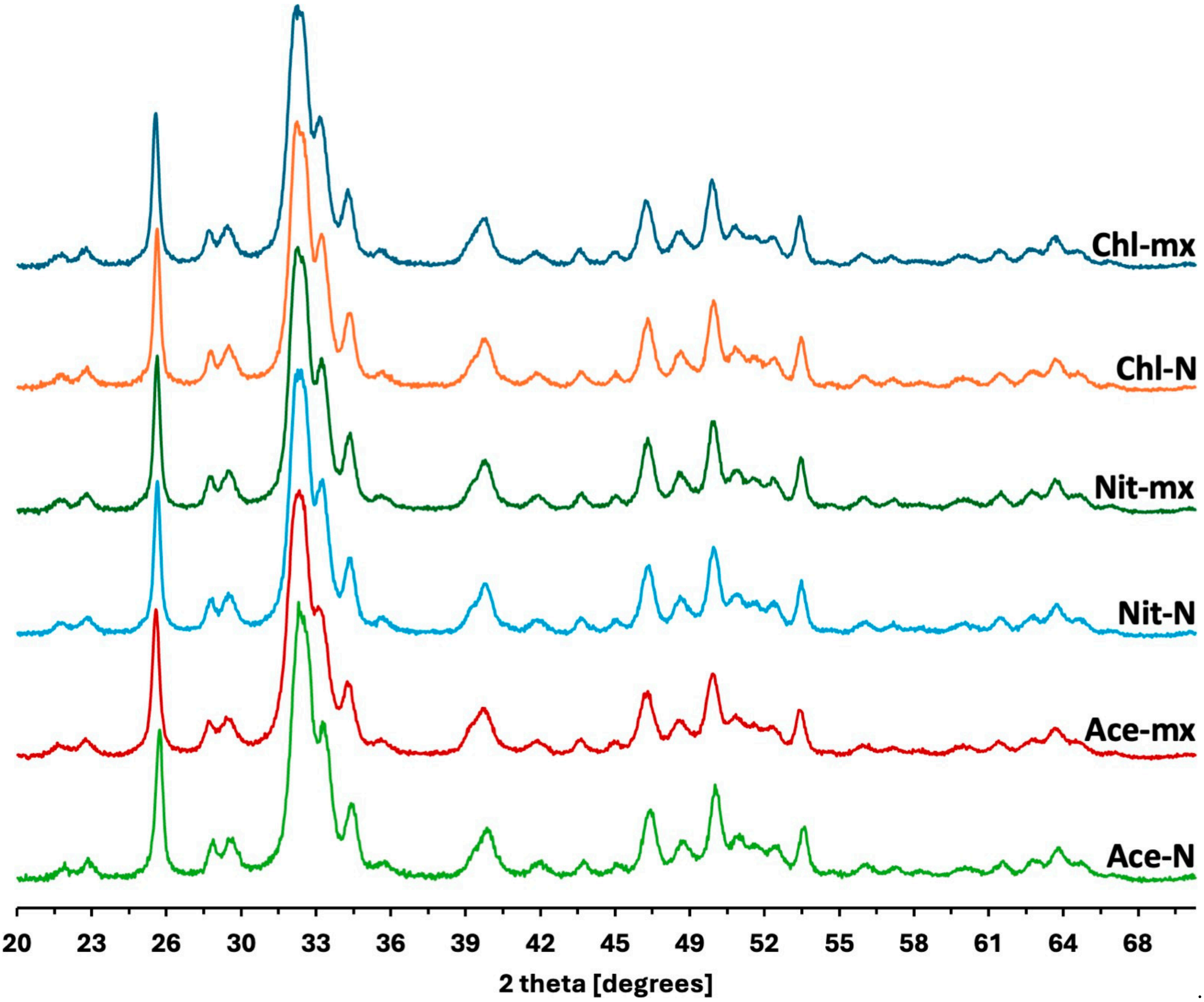

Figure S1. PXRD patterns in conventional form.

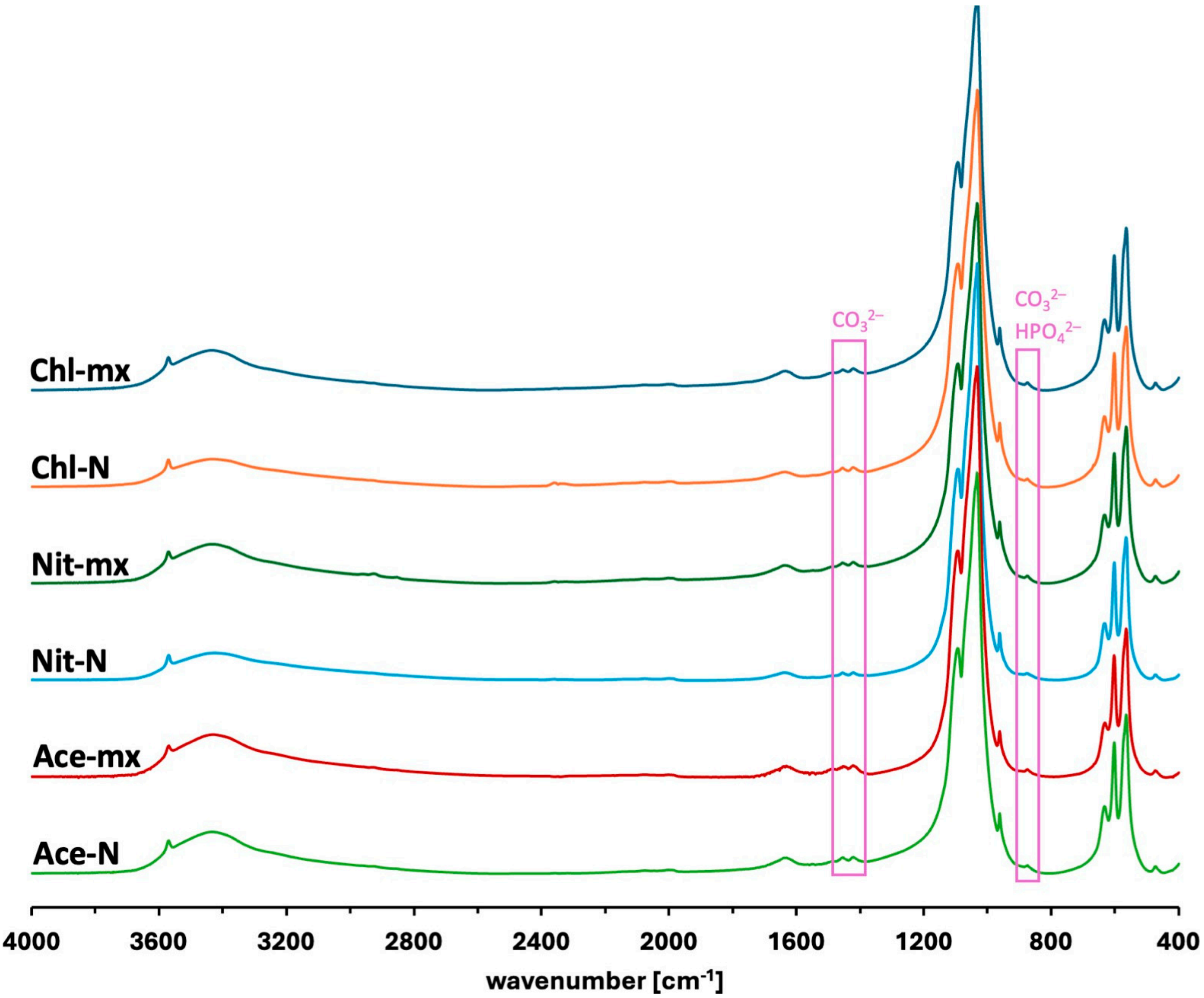

Figure S2. FT-IR spectra in conventional form. Bands corresponding to ions typical of non-stoichiometric hydroxyapatites are highlighted in pink.
